# A Plasmid With Conserved Phage Genes Helps *Klebsiella pneumoniae* Defend Against the Invasion of Transferable DNA Elements at the Cost of Reduced Virulence

**DOI:** 10.3389/fmicb.2022.827545

**Published:** 2022-03-17

**Authors:** Mufeng Cai, Bingchun Pu, Yue Wang, Lin Lv, Chunyu Jiang, Xiaomei Fu, Yan Zhang, Wei Zhao, Ke Dong, Yi Yang, Yangming Liu, Yalu Wei, Zhengyue Zhang, Jianhui Li, Xiaokui Guo, Chang Liu, Jinhong Qin

**Affiliations:** ^1^Department of Microbiology and Immunology, Shanghai Jiao Tong University School of Medicine, Shanghai, China; ^2^Shanghai Institute of Immunology, Shanghai Jiao Tong University School of Medicine, Shanghai, China; ^3^Chinese Center for Tropical Diseases Research, School of Global Health, Shanghai Jiao Tong University School of Medicine, Shanghai, China; ^4^One Health Center, Shanghai Jiao Tong University-The University of Edinburgh, Shanghai, China; ^5^Experiment Teaching Center of Basic Medicine, Shanghai Jiao Tong University School of Medicine, Shanghai, China; ^6^Shanghai Public Health Clinical Center, Shanghai Institute of Phage, Fudan University, Shanghai, China; ^7^NHC Key Laboratory of Parasite and Vector Biology (National Institute of Parasitic Diseases, Chinese Center for Disease Control and Prevention), Shanghai, China

**Keywords:** transferable element, *Klebsiella pneumonia*, plasmid, membrane vesicles (MVs), virulence

## Abstract

*Klebsiella pneumoniae* exhibits extensive phenotypic and genetic diversity. Higher plasmid loads in the cell were supposed to play an key role in its genome diversity. Although some plasmids are widely distributed in *Kp* populations, they are poorly recognized. A plasmid named p2 in strain *Kp1604* was predicted to be an intact prophage like *Salmonella* phage SSU5. However, our study showed that p2 was specifically packaged into membrane vesicles (MVs) rather than phage particles triggered by mitomycin C and subinhibitory concentrations of antibiotics. p2-minus mutant *Kp1604*Δ*p2* did not affect MV production. Compared with *Kp1604*, the capacity of plasmid uptake and the amount of phage burst of *Kp1604*Δ*p2* were improved. Moreover, virulence of *Kp1604*Δ*p2* also increased. Our results indicated that p2 could contribute to the host defense against the invasion of transferable DNA elements at the cost of reduced virulence. Further study on the mechanism will help us understand how it provides adaptive phenotypes to host evolution.

## Introduction

*Klebsiella pneumoniae* (*Kp*) is a gram-negative bacterium belonging to *Enterobacteriaceae*, in which there are also the well-known genera *Salmonella* and *Escherichia* (Wyres et al., 2020). *Kp* can survive in a wide range of host-associated and environmental niches. It can cause a variety of serious hospital-acquired infections, especially in patients with a compromised immune system. Hypervirulent *Kp* (hvKp) infections could cause lethal infections, such as liver abscesses, pneumoniae, meningitis, etc., and have therefore worsened public health worldwide since the early 1980s (Liu et al., 1986). HvKp is an evolving pathotype from classical *Kp* (cKp) due to its acquisition of a cluster of virulence factors encoded on a virulence plasmid and other mobile chromosomally integrated genetic elements (Marr and Russo, 2018; Russo and Marr, 2019). HvKp was rarely resistant to commonly used antimicrobial agents except for being intrinsically resistant to ampicillin for a long period of time after discovery.

In 1996, carbapenem-resistant *Kp* (CRKp) was originally identified in regional outbreaks in the United States (Yigit et al., 2001; Tzouvelekis et al., 2012; Munoz-Price et al., 2013). From then on, multidrug-resistant (MDR) and extremely drug-resistant (XDR) strains continued to emerge as acquisition of plasmids or other transferable genetic elements carrying different antibiotic resistance genes (Munoz-Price et al., 2013; Chen et al., 2014; Wyres and Holt, 2016; Lam et al., 2019). Nowadays, carbapenem-resistant hvKp isolates have emerged (Lam et al., 2019). The emergence of such strains is supposed to be the dissemination of mobile plasmids encoding carbapenemases among HvKp strains or the acquisition of a virulence plasmid by carbapenem-resistant Kp (Wyres et al., 2020). For example, a conjugative plasmid p15 WZ-82_Vir simultaneously encoding antimicrobial resistance (AMR) and virulence determinants was reported, which formed because of the integration of a 100-kb fragment of the hypervirulence plasmid pLVPK into a conjugative IncFIB carbapenem-resistant plasmid (Yang et al., 2019). Such an hv-AMR plasmid could rapidly spread and cause serious infections with extremely limited treatment options, further exacerbating the public health threat posed by Kp (Gao et al., 2020).

*Kp* population has exhibited extensive phenotypic and genetic diversity (Wyres and Holt, 2016; Wyres et al., 2020). However, if only the genes encoded by the core genome were compared, the *Kp* genomics were conserved (Wyres and Holt, 2016; Wyres et al., 2020). The diversity of pan-genome in *Kp* is primarily due to mobile elements that can move frequently by horizontal transfer, including plasmids, phages, integrative and conjugative elements (ICEs), and insertion elements (ISs) (Wyres and Holt, 2016; Wyres et al., 2020). Among the sequenced *Kp* isolates, some strains carried up to 10 different plasmids (Conlan et al., 2016; Wyres et al., 2020). It seems that *Kp* may be particularly permissive for plasmid uptake and/or maintenance, resulting in plasmid loads that are typically greater than those reported for other *Enterobacteriaceae*. Although plasmids are key contributors to the spread of virulence and antibiotic resistance genes, some plasmids that do not encode any known function beyond those may still provide a fitness advantage to their hosts. We sequenced a strain named *Kp1604*, which contained a 5.2 Mb chromosome and 2 plasmids. p1 encoded virulence-related genes, while p2 encoded several conserved phage genes. In this study, we investigated the function and role of plasmid p2 in *Kp1604.*

## Materials and Methods

### Bacterial Strains and Culture Conditions

*Klebsiella pneumoniae* strains, phage and plasmids used in this study are listed in Supplementary Table 1 with their genome accession number if available. The *Kp* strains was sampled from patients as part of routine work in hospital treatment. Competent *Escherichia coli* DH5α was purchased from Takara Biomedical Technology (Beijing) Co., Ltd, Beijing, China. When needed, 50 mg/mL apramycin or spectinomycin was added to the LB broth (1% tryptone, 0.5% yeast extract, 1% NaCl; Sigma-Aldrich, St. Louis, WI, United States).

### Genome Sequencing and Analysis of *Kp1604*

Strain *Kp1604* was grown in LB broth at 37°C with shaking until an OD_600_ of 0.5∼1. DNA from *Kp1604* was extracted and genome sequencing was performed with the PacBio Sequel and Illumina NovaSeq platforms at Shanghai Personal Biotechnology Co., Ltd. The assembly of the whole-genome sequence was carried out by HGAP (Chin et al., 2016) and CANU (Koren et al., 2017). Gene prediction was carried out by GeneMarkS (Besemer et al., 2001).

For further sequence analysis of strain *Kp1604*, the predicted genes were blasted with the VFDB^1^ and CARD^2^ to identify virulence factors and antibiotic resistance genes. Multilocus sequence typing (MLST) and capsular typing by the wzi allele was performed online.^3^ The CRISPR-Cas systems, genomics islands, insertion sequences, prophages and replicons were analyzed online by CRISPRCas Finder,^4^ islandviewer,^5^ ISfinder,^6^ Phaster^7^ and PlasmidFinder 2.1 (Carattoli and Hasman, 2020).

### Bioinformatics Analysis of Plasmid p1 and p2

A comparative plasmid map of p1 (CP085479) in strain *Kp1604* with three known virulence plasmids of phvKp060 (CP034776.1), pK2044 (AP006726.1), and pLVPK (Y378100) was drawn with BLAST Ring Image Generator (BRIG) using pLVPK as a reference sequence (Alikhan et al., 2011).

For comparative analysis of plasmid p2 in strain *Kp1604*, BLASTn search of p2 (CP085480) with the *Kp* strains (Supplementary Table 1) sequenced in this study showed sequence similarity with plasmids of pKp1 (CP086286) and pAF41-1 (JAJGSV000000000). Further, the sequences of *Enterobacteriaceae* plasmids publicly available (2,423 in total) were downloaded from BACTERIAL BIOINFORMATICS RESOURCE CENTER, PATRIC.^8^ The PlasmidFinder 2.1 tools was used to identify replicons in the plasmid sequences (Carattoli and Hasman, 2020). For plasmid cluster analysis, annotation for each plasmid sequence was generated using Prokka (Seemann, 2014) with default parameters. After annotation, 2,426 plasmids were clustered by gene overlap method as following. General Feature Format Version 3 (Gff3) files generated by Prokka were analyzed for gene presence/absence matrix by Roary (Page et al., 2015) with default parameters. To build a plasmid gene presence/absence, matrix in which each entry was 1 if plasmid contained a homolog found in gene cluster or 0 if plasmid did not contain a homolog found in gene cluster. With the matrix, the percentage of gene overlap was calculated by dividing the overlapping gene cluster by all gene clusters of any two plasmid sequences. Then, plasmids were clustered by the Markov cluster algorithm based on gene overlap (van Dongen and Abreu-Goodger, 2012). Network construction Adjacency matrices and network edge lists were created in R. The map of the sequence comparison across plasmid clusters was drawn by Easyfig (Sullivan et al., 2011).

### Antimicrobial Susceptibility Testing and Isolation of Membrane Vesicles

Frozen stock *Kp1604* was grown in LB broth at 37°C overnight. The MICs of chloramphenicol (CHL), tetracycline (TET), ciprofloxacin (CIP) and kanamycin (KAN) were determined by microdilution methods according to the Clinical Laboratory Standards Institute (CLSI), and 1/2 MICs of them used in this study were 4, 4, 2, and 16 μg/mL, respectively. For isolation of membrane vesicles (MVs) from *Kp1604*, overnight cultures were adjusted to a starting OD_600_ of 0.1. Cells were grown until an OD_600_ of 0.6 at 37°C with shaking and then supplemented with 5 μg/mL mitomycin C, 1/2 MIC CHL (4 μg/mL), TET (4 μg/mL), CIP (2 μg/mL), or KAN (16 μg/mL) and cultured for 8 h. Cells cultured without any antibiotics was grown at same conditions as control. Cultured bacterial cells were removed from the supernatant by centrifugation at 8,000 g for 15 min at 4°C. The supernatant was filtered through 0.22 μm pore size filters to remove intact cells and debris. The collected supernatant was further dialyzed against phosphate-buffered saline (PBS) buffer three times for 4 h each time. The MVs were then pelleted through ultracentrifugation at 100,000 g for 1 h at 4°C. The pellets were resuspended in PBS buffer for further experiments. To verify the absence of intact cells in the filtered supernatant, 0.2 mL was plated on LB agar and grown at 37°C for 24 h. The purified MVs was stored at 4°C for further analysis.

### Membrane Vesicles Size and Distribution Determination

The purified MVs from 30 mL cultures of *Kp1604* were dissolved in 0.5 mL PBS. The size (diameter) and size distribution profile of MVs was determined by Zetasizer Nano S equipped with 4.0 mW He-Ne laser (633 nm) (Malvern Instruments Ltd., Worcestershire, United Kingdom).

### Electron Microscopy

The purified MVs was negatively stained with 2% phosphotungstic acid and the morphology was examined by transmission electron microscopy (Tecnai G2 Spirit Biotwin, FEI COMPANY, Hillsboro, QR, United States).

### DNA and Proteome Analysis of Membrane Vesicles

The purified MVs was treated with DNase I Takara Biomedical Technology (Beijing) Co., Ltd, Beijing, China at 37°C for 30 min. To test whether the DNA outside the vesicles was completely removed, 2 μl treated MVs was heated at 100°C for 10 min and 16S rRNA sequence was amplified with primers of 27F and 1492R (Supplementary Table 2). DNA from the treated MVs was extracted using the QIAamp DNA Mini Kit (Qiagen, Venlo Netherlands), following manufacturer’s instructions. Sequencing was performed with the Illumina NovaSeq platform at the Chinese National Human Genome Center at Shanghai. The sequence result of *Kp1604-MV* was deposited in GenBank with accession number OK644452. For PCR verification of DNA contained in MVs, two pairs of primers (p1-1-F and p1-1-R, p1-2-F, and p1-2-R) were designed to amplify p1-1 and p1-2 sequence fragment in p1 (Supplementary Table 2). Two pairs of primers (p2-1-F and p2-1-R, p2-2-F and p2-2-R) were designed to amplify p2-1 and p2-2 sequence fragment in p2 (Supplementary Table 2). 16S rRNA sequences was amplified with primers 27F and 1492R.

For proteome analysis of MVs, the purified MV sample was analyzed on a Thermo Fusion Lumos mass spectrometer connected to an Easy-nLC 1200 *via* an Easy Spray (Thermo Fisher, Waltham, MA, United States) according to the protocol. All MS/MS ion spectra were analyzed using PEAKS 10.0 (Bioinformatics Solutions, Waterloo, ON, Canada) for processing, *de novo* sequencing and database searching. Subcellular localization of the proteins identified in MVs was predicted by Psort (Nakai and Horton, 1999).

### Construction of p2-Minus Strain

To delete p2 from strain *Kp1604*, a two-plasmid CRISPR-Cas system was used as previously described (Wang et al., 2018). Preparation of competent *Kp1604* cells and electroporation were carried out as previously described (Wang et al., 2018). Briefly, temperature-sensitive pCasKP-apr was electroporated into *Kp1604*. The CRISPR–Cas9 spacer plasmid was constructed by cloning the *rep* gene sequence (5′-GAGCAAACCTGAGAAGCCAG-3′) of p2 into pSGKP-spe. The resulting plasmid was electroporated into the pCasKP-apr-harboring strain *Kp1604*. The two-plasmid CRISPR-Cas system was cured as described (Wang et al., 2018). The successful p2-minus mutant *Kp1604△p2* was verified by PCR with primers (p2-1-F and p2-1-R, p2-2-F, and p2-2-R) to amplify p2-1 and p2-2 sequence fragment in p2.

### Efficiency of Plasmid Transformation and Phage Infection

To test the efficiency of plasmid transformation, pSGKP-spe was electroporated into competent *Kp1604* and *Kp1604p2* (Wang et al., 2018). *Kp1604* and *Kp1604△p2* were electroporated without plasmid as controls. Transformation frequency is defined as the number of pSGKP-spe-containing transformants divided by the number of control cells that survived electroporation. We evaluated the efficiency of phage infecting *Kp1604* and *Kp1604△p2* by phage spot plaque tittering method as following. 2 μl aliquots of 10-fold serial dilutions of bacteriophage Φ1209, Φ168R, and Φ 9226R were spotted on bacterial *Kp1604* and *Kp1604△p2* lawns to examine plaque formation. The plaque morphology of bacteriophages Φ1209, Φ168R, and Φ 9226R was observed by the soft-agar overlay method (Luong et al., 2020), 10 μl of serially diluted bacteriophage Φ1209, Φ168R, and Φ 9226R was used to infect 100 μl culture of *Kp1604* and *Kp1604△p2* in a 4 mL overlay of LB containing 0.7% agar. Infection plates were incubated at 37°C for 4∼8 h to observe the phage plaque formation.

### Mouse Infection Studies of *Kp1604* and *Kp1604△p2*

Female 6∼8 weeks old BALB/c mice were purchased from the Shanghai SLAC Laboratory Animal Co., Ltd. All animal protocols were approved by the Animal Ethics Committee in accordance with the guidelines for the use of laboratory animals in China. 13 mice per group were infected intraperitoneally with 100 μl PBS buffer, or doses of 5.0 × 10^6^, 5.0 × 10^7^ CFU of overnight culture of *Kp1604* or *Kp1604△p2* in 100 μl PBS buffer. Three mice each group were euthanized at 24 h after challenge to determine the bacterial loads in organs. Serially diluted homogenates of blood, livers, lungs were plated on LB agar and incubated at 37°C overnight for quantification of CFU. The mortality rate of ten mice each group was recorded for 96 h after challenge.

### Statistical Analyses

Survival curves of mice were generated using GraphPad Prism 9. Statistical analyses of the efficiency of plasmid transformation and bacterial loads in various organs of mice were performed by the Mann–Whitney *U*-test (^∗^*p* < 0.05, ^∗∗^*p* < 0.01, ^∗∗∗^*p* < 0.001).

## Results

### Genome Analysis of *Kp1604*

*Klebsiella pneumoniae* strain *Kp1604* was isolated from a patient’s blood sample. The genome sequence showed that *Kp1604* contained a circular chromosome of 5,205,350 bp with a GC content of 57.58% and two plasmids (p1 and p2) of 144,892 bp and 109,308 bp with GC contents of 49.69 and 48.93%, respectively (Table 1 and Supplementary Figure 1). *Kp1604* belonged to ST412 based on multilocus sequence typing (MLST) and the K57 serotype based on capsular typing by the wzi allele.

**TABLE 1:** General features of the *Kp1604* chromosomes.

Features	Chromosome	Plasmid	
		p1	p2
Size (bp)	5,205,350	144,892	109,308
GC content	57.58%	49.69%	48.93%
No. of ORFs	4,855	172	118
Coding percentage	86.46%	81.40%	86.56%
Number of rRNAs	25	0	0
Number of tRNAs	86	0	1
Predicted intact prophage	0	0	1
Genomic islands	21	–	–
Insertion sequence	41	82	0
Replicon	–	IncFIB	IncFIB

The genome sequence of plasmid p1 encoded five virulence-related genes, including *rmpA, iroB, iroC, iroD* and *iroN*. Genome sequence alignment of p1 with pLVPK, a well-known virulence plasmid, showed that they shared regions of homology (Supplementary Figure 2). These results suggested that p1 may be a virulence-related plasmid. The DNA sequence of p2 encoded 118 putative genes, 103 of which were predicted to phage-related genes by Phaster, including phage structure genes, integrase, terminase, etc. (Supplementary Table 3). p2 was predicted to be an intact prophage by Phaster, similar to *Salmonella* phage SSU5 in *Salmonella enterica* serovar Typhimurium rough strain (Kim et al., 2012). Whole-genome sequence analysis of *Kp1604* identified 21 genomic islands but none of the phage-related sequences except p2 (Supplementary Table 4). A BLAST search of the p2 sequence against the CARD and VFDB databases showed that p2 did not carry any virulence-related or AMR-related genes.

### Widely Distribution of p2-Like Plasmid in *Enterobacteriaceae*

**FIGURE 1:**
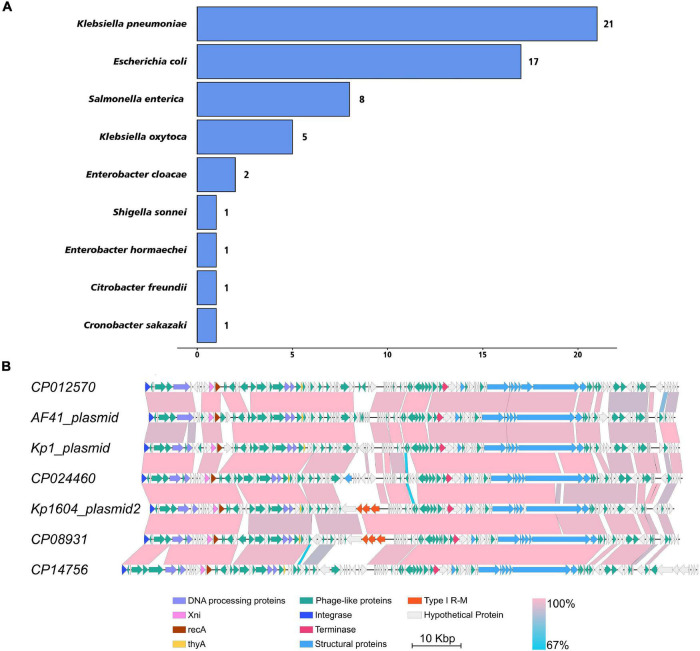
The distribution of p2-like plasmids in *Enterobacteriaceae* and sequence alignment of p2-like plasmids. **(A)** The distribution of p2-like plasmids in *Enterobacteriaceae*. A total of 2,426 plasmid sequences were downloaded and clustered. 57 of them were clustered as p2-like plasmids, which were mainly distributed in *Klebsiella pneumoniae, Escherichia coli, Salmonella enterica, and Klebsiella oxytoca.* **(B)** Sequence alignment of the complete genome of selected p2-like plasmids. The nucleotide identity of the homologous regions (percentage) is indicated in color; the scale is shown below. Known functional homologous genes are shown below.

To investigate the distribution of this kind of plasmid, we downloaded 2,423 plasmid sequences of *Enterobacteriaceae* deposited in the Patricbrc database and three plasmid sequences of *Kp* deposited in GenBank. Plasmid cluster analysis showed that cluster 1 was an MDR plasmid cluster, including pSWU01 and pKP048 (Supplementary Figure 3); cluster 11 was virulent plasmid cluster, including pLVPK and pNTUH-K2044 (Supplementary Figure 3). p2 clustered to cluster 13, which contained 57 plasmids (Supplementary Figure 3 and Figure 1A). The PlasmidFinder analysis of cluster 13 showed they all belonged to same replicon of IncFIB. Of them, 21, 17, 8, and 5 plasmids were distributed in *Klebsiella pneumoniae, Escherichia coli, Salmonella enterica* and *Klebsiella oxytoca*, respectively (Figure 1A). Notably, all p2-like plasmid-harboring strains were isolated from human samples where information about isolation was available. Further alignment of selected p2-like plasmids showed that they all encoded several phage-related proteins, integrase, terminase, recA, Xni and thyA (Figure 1B).

### p2 DNA Specifically Packaged Into Membrane Vesicles in Response to Mitomycin C and Subinhibitory Concentrations of Antibiotics

**FIGURE 2:**
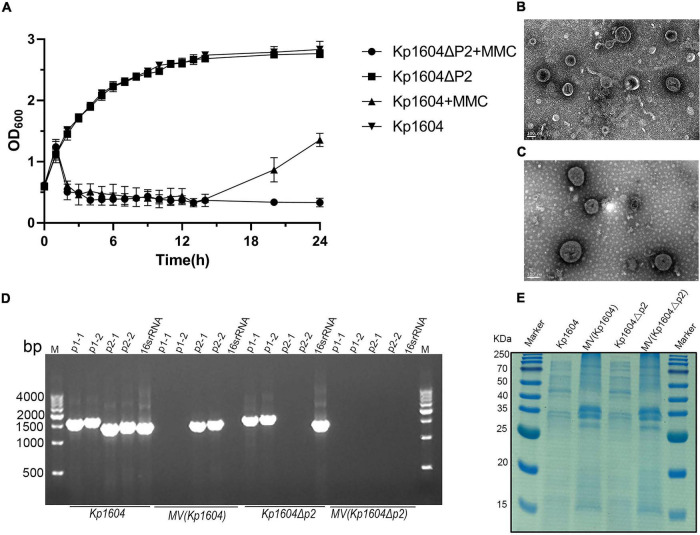
MVs analysis from *Kp1604* and *Kp1604*Δ*p2*. **(A)** The growth of *Kp1604* and *Kp1604*Δ*p2* with or without mitomycin C. Transmission electron microscopy photograph of MV products from *Kp1604* **(B)** and *Kp1604*Δ*p2* **(C)** with mitomycin C induction. Scale bar of 100 nm is shown in the images. **(D)** PCR amplification of sequence fragment of p1, p2 and 16S rRNA. As indicated below, *Kp1604* and *Kp1604*Δ*p2* represent DNA extracted from whole cell lysates; MV(*Kp1604*) and MV(*Kp1604*Δ*p2)* represent DNA extracted from purified supernatants treated with DNase I. As indicated above, p1-1 and p1-2 represent specific sequence fragment in p1 plasmid amplified with primers p1-1-F and p1-1-R, p1-2-F and p1-2-R; p2-1 and p2-2 represent specific sequence fragment in p2 plasmid amplified with primers p2-1-F and p2-1-R, p2-2-F and p2-2-R; 16S rRNA was amplified with primers 27F and 1492R. **(E)** SDS–PAGE analysis of protein content in MVs and whole cell lysates of *Kp1604* and *Kp1604*Δ*p2. Kp1604* and *Kp1604*Δ*p2* represent whole cell lysates, and MV(*Kp1604*) and MV(*Kp1604*Δ*p2)* represent purified supernatants from *Kp1604* and *Kp1604*Δ*p2* triggered with mitomycin C.

Stresses such as UV light and mitomycin C are known to stimulate the SOS response and induce prophage particles (Du Toit, 2019). We therefore evaluated the impact of mitomycin C exposure on strain *Kp1604* to investigate whether plasmid p2 could be induced as a phage. After supplementation of mitomycin C, the growth of bacteria began to decline after 2 h (Figure 2A). TEM analysis of the purified supernatant revealed round particles sized from 50 to 300 nm (Figure 2B). p2 DNA was confirmed to specifically package into particles, as identified by both DNA sequence (accession number OK644452) and PCR amplification (Figure 2D). However, when the collected particles were spotted on 14 different *Kp* strains, no phage plaque was observed. SDS–PAGE analysis showed that the protein profiles of purified particles were different from those of bacterial lysates (Figure 2E). A proteomic analysis showed that a total of 221 proteins were identified, and OmpC and OmpA were the major components, accounting for 1.86 and 1.55%, respectively, which is consistent with the band pattern of SDS–PAGE (Supplementary Table 5). There were 81, 86, 40, and 14 identified proteins predicted to be located in the cytoplasm, inner membrane, outer membrane and periplasmic space, respectively (Supplementary Table 5). Combined with the identified proteins, this structure was more similar to MVs than phages. Therefore, it suggested that *Kp1604* secreted p2 DNA-containing MVs after induction by mitomycin C.

**FIGURE 3:**
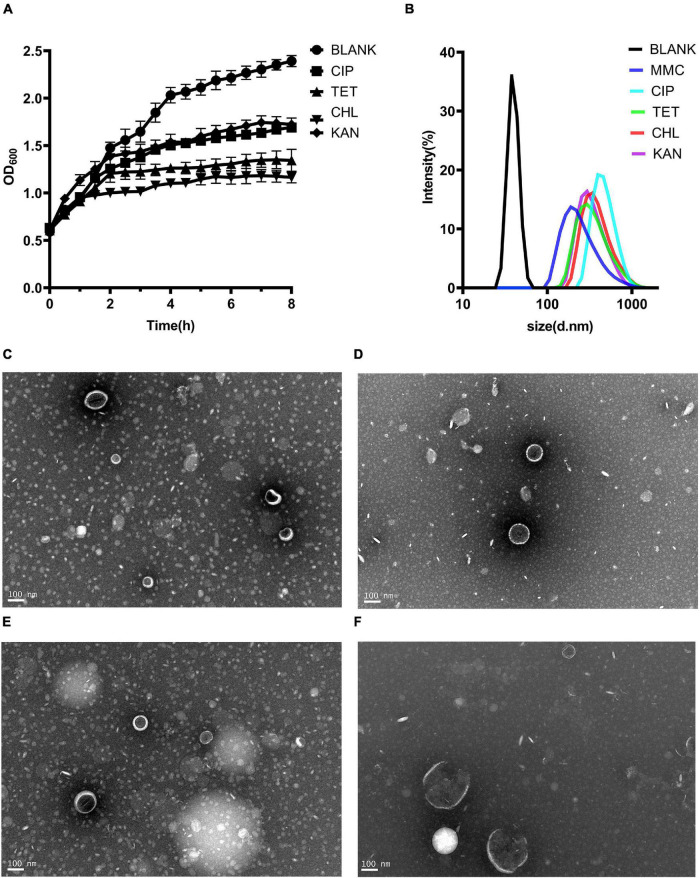
Production of MVs from *Kp1604* cultured under antibiotic stress conditions. **(A)** Bacterial growth of *Kp1604* cultured with 1/2 MIC of ciprofloxacin (CIP), tetracycline (TET), chloramphenicol (CHL) and kanamycin (KAN). **(B)** Size and distribution of MVs. Single particle tracking analysis of MVs isolated from *Kp1604* after treatment with 1/2 MIC of CHL **(C)**, TET **(D)**, CIP **(E)**, and KAN **(F)**, as compared to non-treated (BLANK). Transmission electron microscopy photograph of MVs produced by *Kp1604* cultured with 1/2 MIC of CHL **(B)**, TET **(C)**, CIP **(D)**, and KAN **(E)**. Scale bars of 100 nm are shown in the images.

MVs are thought to be produced when bacteria are under environmental stress (Toyofuku et al., 2019). Four kinds of sub-inhibitory concentration antibiotics, chloramphenicol, ciprofloxacin, tetracycline and kanamycin were selected. Although the growth of bacteria did not decline significantly under antibiotic stress, it was inhibited (Figure 3A). Single particle tracking (SPT) analysis revealed that major particles in the induced supernatant were 100∼300 nm in size (Figure 3B). TEM analysis further confirmed there were round vesicles of the same size (Figures 3C–F).

### Deletion of p2 Improved the Acquisition of Transferable Elements in *Kp1604*Δ*p2*

To demonstrate that MV production was not p2-related, we deleted p2 from *Kp1604* and obtained the plasmid-minus mutant strain *Kp1604*Δ*p2*. Deletion of p2 did not affect the growth of *Kp1604*Δ*p2* compared with *Kp1604* (Figure 2A). MVs were produced by *Kp1604*Δ*p2* with mitomycin C induction, similar to *Kp1604* (Figure 2C). Further protein analysis by SDS–PAGE showed negligible differences in the protein composition between MVs from *Kp1604* and the plasmid-minus mutant *Kp1604*Δ*p2* (Figure 2E). This experiment further demonstrated that *Kp1604* produced MVs rather than phages triggered by mitomycin C.

**FIGURE 4:**
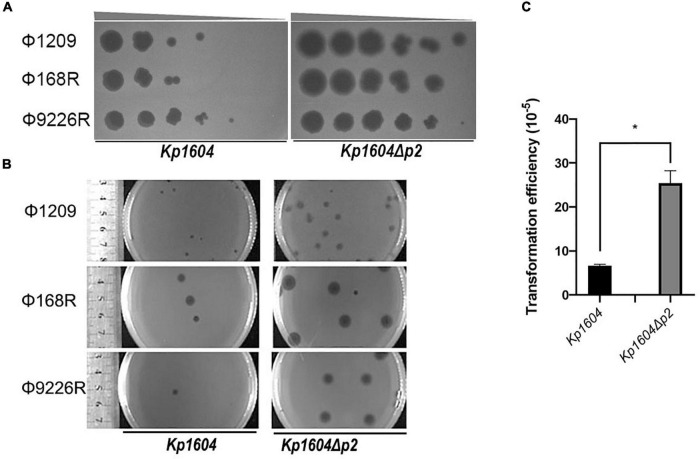
Deletion of p2 from *Kp1604* affected the acquisition of transferable elements. **(A)** The efficiencies of phage propagation assays. Ten-fold serial dilutions of phages Φ1209, Φ168R, and Φ9226R were spotted on lawns of *Kp1604* and *Kp1604*Δ*p2*, respectively, from 10^–3^ to 10^–8^ serial dilutions as indicated. **(B)** Phage plaque diameter of Φ1209, Φ168R, and Φ9226R infecting *Kp1604* and *Kp1604*Δ*p2*. **(C)** Transformation efficiencies of strains *Kp1604* and *Kp1604*Δ*p2* by electroporation with plasmid pSGKP-spe. Transformation efficiency was defined as the number of pSGKP-spe-containing transformants divided by the number of control cells that survived electroporation, *p < 0.05.

To determine whether p2 could inhibit other phage infections against *Kp1604*, the efficiencies of plating (EOP) of three lytic phages of Φ1209, Φ168R, and Φ9226R were spotted on *Kp1604* and *Kp1604*Δ*p2* lawn with serial dilutions. We observed the same pattern with three lytic phages: their EOP on *Kp1604* was reduced by 1∼2 orders of magnitude compared to the mutant *Kp1604*Δ*p2* (Figure 4A). In addition, the phage plaques on *Kp1604*Δ*p2* were significantly larger in diameter than those on *Kp1604* (Figure 4B). These results suggest that p2 could mediate host defense against phage infection. Plasmid acquisition was also investigated using plasmid pSGKP-spe transformation into strains *Kp1604* and *Kp1604*Δ*p2*. The plasmid acquisition efficiency of *Kp1604*Δ*p2* was significantly higher than that of *Kp1604* (Figure 4C). Thus, the presence of p2 could help host defend against foreign DNA invasion.

### Deletion of p2 Increased Bacterial Virulence of *Kp1604*Δ*p2*

**FIGURE 5:**
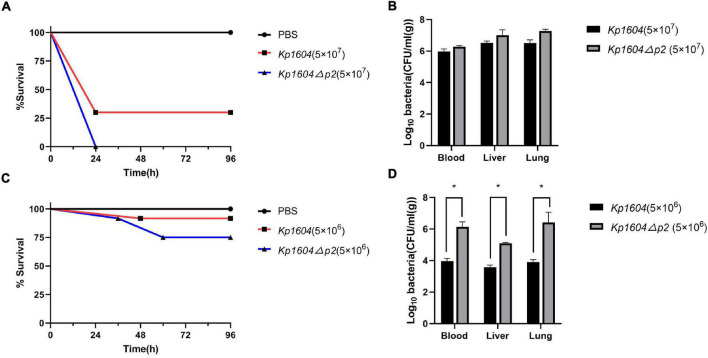
Virulence potential of *Kp1604* and *Kp1604*Δ*p2*. Percent survival of mice with an inoculum of 5 × 10^7^ CFU **(A)** and 5 × 10^6^ CFU **(C)**. Strains of *Kp1604* and *Kp1604*Δ*p2* were monitored with intraperitoneal injection in 10 mice per group. Bacterial loads in blood, liver, and lung with an inoculum of 5 × 10^7^CFU **(B)** and 5 × 10^6^ CFU **(D)**. Three mice each group were euthanized at 24 h after challenge. Blood, livers, and lungs were aseptically homogenized. For the bacterial load analysis, serially diluted homogenates were cultured overnight for counting. *p < 0.05.

The virulence potentials of strains *Kp1604* and *Kp1604*Δ*p2* were determined in mouse infection models with intraperitoneal injection. To our surprise, the virulence of *Kp1604*Δ*p2* increased significantly with an inoculum of 5 × 10^7^°CFU, resulting in 100% mortality at 24 h, whereas only 70% mortality was observed for *Kp1604* (Figure 5A). There was no significant difference in bacterial load in various tissues of the mice when challenged with *Kp1604* and *Kp1604*Δ*p2* at a dose of 5 × 10^7^ CFU (Figure 5B). When challenged with the lower dose of 5 × 10^6^ CFU, 10% mortality was observed for *Kp1604* and *Kp1604*Δ*p2* at 48 h, and 10 and 25% mortality was observed for *Kp1604* and *Kp1604*Δ*p2*, respectively, at 60 h (Figure 5C). However, the bacterial loads in the liver, lung and blood of mice at 24 h after challenge at the lower dose of 5 × 10^6^CFU showed that the bacterial load of the *Kp1604*Δ*p2* strain was significantly higher than that of the *Kp1604* strain (Figure 5D). These data implied that plasmid p2 decreased the virulence of *Kp1604* in mice.

## Discussion

Plasmids have an important role in bacterial evolution by transferring beneficial traits within and between species of bacteria, potentially contributing to host advantage fitness (Rodriguez-Beltran et al., 2021). Many diverse plasmids have been sequenced from *Kp*, among which virulence plasmids and AMR plasmids have been widely studied (Ernst et al., 2020; Wyres et al., 2020; Yang et al., 2020). Although some plasmids are widely distributed in *Kp* populations, their beneficial contribution to host was far from studied.

In this study, we isolated a virulent *Kp* strain harboring two plasmids inside the cell. p2 was predicted to be an intact prophage. Typically, phages integrate into bacterial chromosomes to be prophages. However, numerous phage plasmids have also been reported as extrachromosomal plasmids based on their encoding proteins homologous to both phage-related and plasmid-related proteins (Ikeda and Tomizawa, 1968; Zhang et al., 2017; Gilcrease and Casjens, 2018; Galetti et al., 2019; Pfeifer et al., 2021). Although plasmid prophages such as P1 and P6 of *Escherichia coli* can be both maintained as plasmids and converted to viral particles for release, most of the others cannot be released as viral particles (Ikeda and Tomizawa, 1968; Gilcrease and Casjens, 2018). The conversion controls between plasmid replication and bacteriophage lytic cycle is highly sophisticated, and the absence of any of the key regulators may lead to conversion failure (Sternberg and Austin, 1981). Although a total of 103 genes in p2 was predicted to encode conserved phage proteins, p2 still could not be induced as phage. Therefore, it suggested that p2 was still a plasmid that carried many phage-like elements.

Membrane vesicles are nanoparticles sized from 20 to 400 nm in diameter originating from blebbing of the outer membrane of bacteria (Toyofuku et al., 2019). The structure and composition of MVs secreted by different bacteria are different. It was reported that proteins, nucleic acids, toxins could be packaged into MVs (Schwechheimer and Kuehn, 2015; Gilmore et al., 2021). MVs play a significant role in biological processes including virulence, horizontal gene transfer (Chatterjee et al., 2017; Bielaszewska et al., 2020), export of cellular metabolites (Orench-Rivera and Kuehn, 2016), phage infection and cell-to-cell communication (Zingl et al., 2020). In our study, mitomycin C and sub-inhibitory concentration of antibiotics could trigger p2-DNA contained MVs secretion in strain *Kp1604*. The function of P2 specifically packaged into MVs should be further investigated.

This study confirmed that p2 could contribute to defense against phage infections and affect uptake of plasmids. Bacteria have evolved numerous defense mechanisms against mobile genetic elements (MGEs) invasion, including restriction modification (RM) systems, CRISPR-Cas systems, abortive infection (Abi) systems and others (Samson et al., 2013). The RM system is one of the innate immune systems which limits incorporation of MGEs. RM system encoded by p2 was inferred to defense MGEs uptake in *Kp1604*. On the other hand, plasmid encodes genes to ensure itself replication, maintenance, and transmission, which are often considered disadvantageous to the host (Rodriguez-Beltran et al., 2021). Our results showed that the mortality of *Kp1604* was decreased compared with that of *Kp1604*Δ*p2* when challenged in a mouse model. It seemed that *Kp1604* reduced virulence at cost to maintenance of p2. Plasmids are widely distributed and highly diverse in the *Kp* population (Wyres et al., 2020; Yang et al., 2020). Further study on the mechanism of providing adaptive phenotypes to bacterial hosts will help us understand new contributions of plasmids to host evolution.

## Data Availability Statement

The datasets presented in this study can be found in online repositories. The names of the repository/repositories and accession number(s) can be found in the article/Supplementary Material.

## Ethics Statement

The animal study was reviewed and approved by the Shanghai Jiao Tong University School of Medicine.

## Author Contributions

MC, BP, CL, and JQ contributed to the study design and wrote the manuscript. MC, BP, YW, LL, CJ, YZ, WZ, KD, YY, YL, YLW, ZZ, JL, XG, CL, and JQ contributed to experiment and data collection. JQ, CL, BP, and CJ did the data analyses. All authors contributed to data interpretation, provided critical revision of the manuscript, approved the final manuscript, and agreed to submit for publication.

## Conflict of Interest

The authors declare that the research was conducted in the absence of any commercial or financial relationships that could be construed as a potential conflict of interest.

## Publisher’s Note

All claims expressed in this article are solely those of the authors and do not necessarily represent those of their affiliated organizations, or those of the publisher, the editors and the reviewers. Any product that may be evaluated in this article, or claim that may be made by its manufacturer, is not guaranteed or endorsed by the publisher.

## Footnotes

1
http://www.mgc.ac.cn/VFs/

2
https://card.mcmaster.ca/

3
https://bigsdb.pasteur.fr

4
http://crispr.i2bc.paris-saclay.fr/Server/

5
https://www.pathogenomics.sfu.ca/islandviewer/

6
https://www-is.biotoul.fr/

7
http://phaster.ca/

8
https://www.patricbrc.org/
