# Supplementary material for: A Plasmid With Conserved Phage Genes Helps Klebsiella pneumoniae Defend Against the Invasion of Transferable DNA Elements at the Cost of Reduced Virulence
Source: Front Microbiol. 2022 Mar 17;13:827545. doi: 10.3389/fmicb.2022.827545 (PMC8969562; doi:10.3389/fmicb.2022.827545)

Figure S1. The genome of Kp1604. Chromosome and plasmids were present in circle plot using the circlize R package. The feature of each circle was shown by legend as indicated.


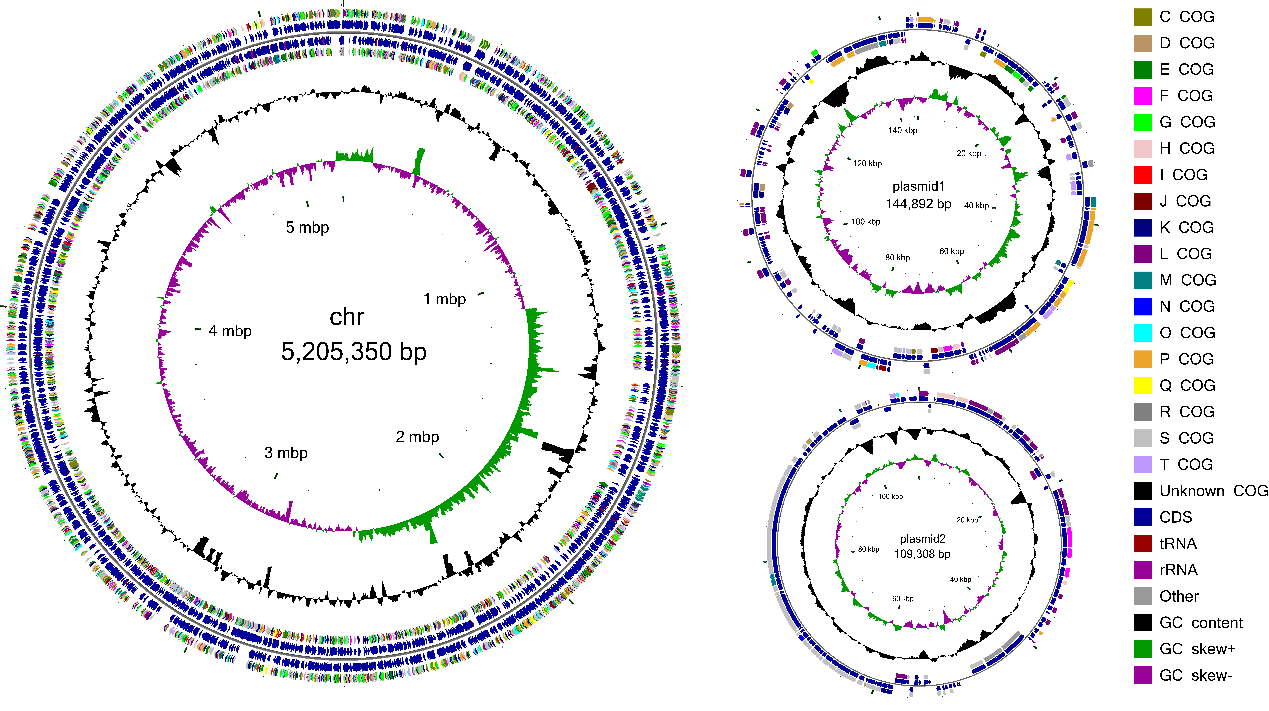


Figure S2. Sequence comparative map of plasmid p1 and three known virulence plasmids (phvKp060, CP034776.1; pK2044, AP006726.1; pLVPK, AY378100). The pLVPK sequence was used as reference.


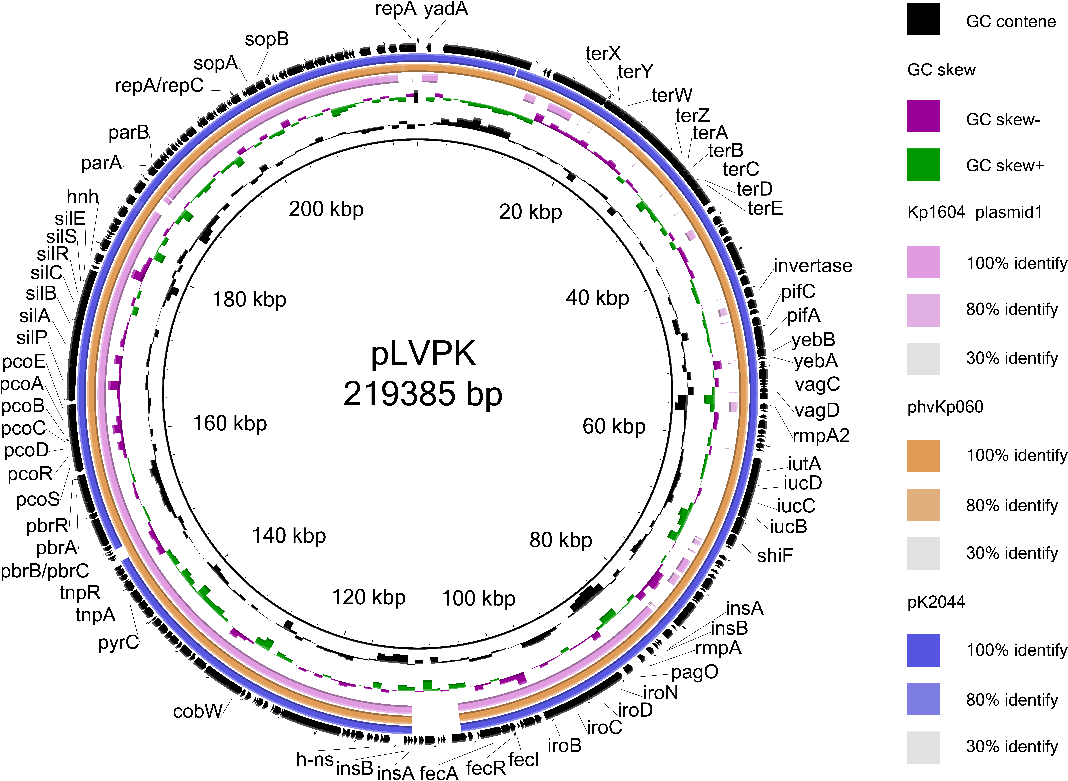


Figure S3. Plasmid clusters in *Enterobacteriaceae*. The plasmids were clustered by MCL based on proteins overlap matrix. The result were shown by heatmap using pheatmap R package, with colour shade corresponding to the fraction of proteins overlap. Cluster1 belonged to MDR family which included well known plasmids as SWU01 and KP048; Cluster 11 belonged to virulent family which included pLVPK and pK2044; Cluster13 belonged to p2-like plasmids cluster which referred in this study.


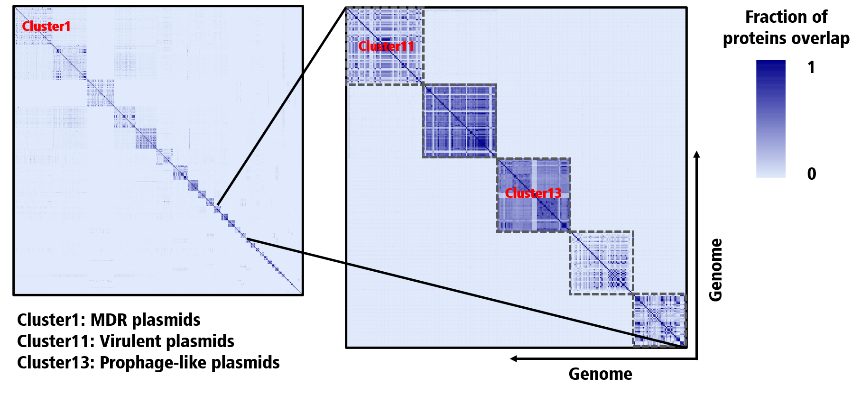

Supplement: Supplementary file 1 [file Data_Sheet_1.DOCX]
