# Supplementary material for: A Plasmid With Conserved Phage Genes Helps Klebsiella pneumoniae Defend Against the Invasion of Transferable DNA Elements at the Cost of Reduced Virulence
Source: Front Microbiol. 2022 Mar 17;13:827545. doi: 10.3389/fmicb.2022.827545 (PMC8969562; doi:10.3389/fmicb.2022.827545)
Supplement: Supplementary file 2 [file Table_1.DOCX]

Table S1 strains, plasmids and phages used in this study

|  | type | GenBank No. | Sources |
| --- | --- | --- | --- |
| Strains |  |  |  |
| E. coli DH5α | *-* | - | Takara Biomedical Technology (Beijing) Co., Ltd |
| Kp1604 | *K. pneumoniae* ST412 | CP0865478, CP0865479, CP0865478, | This study |
| Kp1 | *K. pneumoniae* ST1017 | CP086285 | This study |
| Kp36 | *K. pneumoniae* ST11 | CP047192 | This study |
| Kp168 | *K. pneumoniae* ST11 | JAKCWX000000000 | This study |
| Kp1231 | *K. pneumoniae* ST15 | SAMN13324146 | Qin et al., 2020 |
| Kp5152 | *K. pneumoniae* ST11 | CP090462 | This study |
| Kp6377 | *K. pneumoniae* ST11 | JAKCWY000000000 | This study |
| Kp7450 | *K. pneumoniae* ST15 | CP090468 | This study |
| Kp9226 | *K. pneumoniae* ST147 | JAKCWZ000000000 | This study |
| HC001KP3 | *K. pneumoniae* ST34 | JAKCXA000000000 | This study |
| HC001KP5 | *K. pneumoniae* ST5731 | JAKCXB000000000 | This study |
| HC001KP6 | *K. pneumoniae* ST70 | JAKCXC000000000 | This study |
| AF001KP3 | *K. pneumoniae* ST36 | JAKCXD000000000 | This study |
| AF002KP4 | *K. pneumoniae* ST1213 | JAKCXE000000000 | This study |
| AF004KP1 | *K. pneumoniae* ST4982 | JAJGSV000000000 | This study |
| Plasmid |  |  |  |
| pSGKP-spe | *genome editing plasmid of K. pneumoniae* | - | Wang Y et al., 2018(Ref 24.) |
| pCasKP-apr | *genome editing plasmid of K. pneumoniae* | - | Wang Y et al., 2018(Ref 24.) |
| Phage |  |  |  |
| Ф1209 |  |  | This study |
| Ф168R |  |  | This study |
| Ф 9226R |  |  | This study |
