# Supplementary material for: A Plasmid With Conserved Phage Genes Helps Klebsiella pneumoniae Defend Against the Invasion of Transferable DNA Elements at the Cost of Reduced Virulence
Source: Front Microbiol. 2022 Mar 17;13:827545. doi: 10.3389/fmicb.2022.827545 (PMC8969562; doi:10.3389/fmicb.2022.827545)
Supplement: Supplementary file 3 [file Table_2.DOCX]

| Primer | Sequence | product Length  (bp) | target replicons | Location |
| --- | --- | --- | --- | --- |
| p1-1-F | TGCCATCAGTGCCGACAGT | 1,700 | amplification of p1-1 fragment at p1 | 18,789-20,488 |
| p1-1-R | CCAGACAGCGTGAACCAGTTAA |  |  |  |
| p1-2-F | TGATGAAGCCGCAGCACTTG | 1,728 | amplification of p1-2 fragment at p1 | 77,687-79,414 |
| p1-2-R | CCGCAACACCAGAAGAATACCA |  |  |  |
| p1-2-R | AAGAGCCACGAACAAGCGAAT | 1,500 | amplification of p2-1 fragment at p2 | 12,119-13,618 |
| p2-1-R | GCAAGACGACCAACCAGCAT |  |  |  |
| p2-2-F | CCAGAACCAATGATGCCAACCT | 1,579 | amplification of p2-2 fragment at p2 | 57,201-58,780 |
| p2-2-R | TCCAGACGCTTCGCCTTCA |  |  |  |
| 27F | AGAGTTTGATCMTGGCTCAG | 1,466 | Chromosome | 16s rRNA |
| 1492R | GGTTACCTTGTTACGACTT |  |  |  |

Table S2 Primers used in this study
