# Supplementary material for: A Plasmid With Conserved Phage Genes Helps Klebsiella pneumoniae Defend Against the Invasion of Transferable DNA Elements at the Cost of Reduced Virulence
Source: Front Microbiol. 2022 Mar 17;13:827545. doi: 10.3389/fmicb.2022.827545 (PMC8969562; doi:10.3389/fmicb.2022.827545)
Supplement: Supplementary file 5 [file Table_4.DOCX]

Table S4 Island sequence identified on *Kp1604* chromosome

| ISL Name | location | | | length (bp) | | No. of ORFs | Predicted functions |
| --- | --- | --- | --- | --- | --- | --- | --- |
|  | start | | end |  |  |  |  |
| ISL1 | 322,469 | 328,811 | | 6,342 | 4 | | unknown |
| ISL2 | 466,073 | 471,860 | | 5,787 | 9 | | unknown |
| ISL3 | 1,372,832 | 1,382,399 | | 9,567 | 8 | | unknown |
| ISL4 | 1,939,548 | 1,945,497 | | 5,949 | 5 | | virulent relative island |
| ISL5 | 2,180,214 | 2,186,489 | | 6,275 | 8 | | unknown |
| ISL6 | 2,198,945 | 2,208,375 | | 9,430 | 10 | | unknown |
| ISL7 | 2,221,333 | 2,239,225 | | 17,892 | 13 | | unknown |
| ISL8 | 2,486,457 | 2,491,072 | | 4,615 | 6 | | unknown |
| ISL9 | 2,493,642 | 2,500,980 | | 7,338 | 8 | | unknown |
| ISL10 | 2,504,510 | 2,509,805 | | 5,295 | 9 | | unknown |
| ISL11 | 2,532,561 | 2,537,797 | | 5,236 | 7 | | unknown |
| ISL12 | 2,654,100 | 2,658,659 | | 4,559 | 6 | | unknown |
| ISL13 | 2,835,721 | 2,844,189 | | 8,468 | 10 | | unknown |
| ISL14 | 2,880,977 | 2,886,937 | | 5,960 | 8 | | unknown |
| ISL15 | 2,887,888 | 2,892,452 | | 4,564 | 5 | | unknown |
| ISL16 | 3,026,918 | 3,031,314 | | 4,396 | 5 | | unknown |
| ISL17 | 3,055,367 | 3,061,233 | | 5,866 | 8 | | unknown |
| ISL18 | 4103097 | 4,113,094 | | 9,997 | 11 | | unknown |
| ISL19 | 4,380,890 | 4,389,169 | | 8,279 | 8 | | unknown |
| ISL20 | 4,566,602 | 4,570,937 | | 4,335 | 4 | | unknown |
| ISL21 | 4,590,441 | 4,594,625 | | 4,184 | 2 | | unknown |
